# The effect of positive mood induction on reducing reinstatement fear: Relevance for long term outcomes of exposure therapy

**DOI:** 10.1016/j.brat.2015.05.016

**Published:** 2015-08

**Authors:** Tomislav D. Zbozinek, Emily A. Holmes, Michelle G. Craske

**Affiliations:** a University of California, Los Angeles, Department of Psychology, 1285 Franz Hall, Los Angeles, CA 90025, USA; b MRC Cognition & Brain Sciences Unit, 15 Chaucer Road, Cambridge CB2 7EF, England, UK; c Department for Clinical Neuroscience, Karolinska Institutet, K8, Psychology, 171 77 Stockholm, Sweden

**Keywords:** Fear conditioning, Exposure therapy, Anxiety, Reinstatement, Return of fear, Conditional stimulus valence

## Abstract

While exposure therapy is effective in treating anxiety, fear can return after exposure. Return of fear can be understood through mechanisms of extinction learning. One form of return of fear is reinstatement, or, the fear that results from an unsignaled unconditional stimulus (US) presentation after extinction. Though the conditional response (CR; e.g., fear) typically reduces during extinction, the excitatory conditional stimulus (CS+) valence remains negative. The more negative the CS+ valence after the end of extinction, the greater the fear at reinstatement. The current study evaluated the degree to which positive mood induction (positive imagery training; PIT) compared to control (positive verbal training; PVT) before extinction a) decreased CS+ negative valence during extinction and b) reduced reinstatement fear. Compared to PVT, PIT a) increased positive affect, b) decreased post-extinction CS+ negative valence, and c) reduced reinstatement responding as measured by eye blink startle reflex (when shock was used at reinstatement) and self-report fear (regardless of reinstatement US type). Results suggest that increasing positive affect prior to exposure therapy could reduce relapse through reinstatement.

## Highlights

• Post-extinction CS+ valence is a known predictor of reinstatement CS+ fear.
• Pre-extinction positive mood induction increased post-extinction CS+ valence.
• Pre-extinction positive mood induction decreased reinstatement CS+ fear.
• Results suggest positive mood induction prior to exposure could reduce relapse in anxiety disorders.

## 1 Introduction

Exposure therapy is well-established as an effective therapeutic strategy for anxiety disorders (Hofmann & Smits, 2008; In-Albon & Schneider, 2007). However, a number of individuals experience a return of fear following successful conclusion of treatment (Craske & Mystkowski, 2006; Rachman, 1989). Thus, there is a need to understand the mechanisms responsible for return of fear and to develop interventions that reduce its occurrence and enhance long-term treatment gains. In the model of exposure therapy, return of fear is understood as reactivation of conditional threat associations that compete with the non-threat-based associations developed through extinction (Hermans, Craske, Mineka, & Lovibond, 2006). The purpose of the current study is to evaluate one possible method (i.e., positive mood induction before extinction) of reducing return of fear following extinction.

Models of extinction emphasize inhibitory learning mechanisms (Bouton, 1993; Wagner, 1981), although additional mechanisms, such as habituation, may also be involved (Myers & Davis, 2007). Within a classical conditioning approach, the original conditional stimulus (CS)/unconditional stimulus (US) association learned during acquisition of threat responding^1^ is not erased during extinction, but rather is left intact while a new, secondary CS+/NoUS inhibitory association develops (e.g., Bouton, 1993; Bouton & King, 1983). This means that individuals have two memories of the CS+: one in which it predicts an aversive event and a separate memory in which it predicts no aversive event. The relative strength between these two memories determines how much threat responding occurs. In these studies, a CS+ is associated with the occurrence of the US, whereas a CS− is associated with the absence of the US. The inhibitory association is dependent on both the CS+ and the context in which the CS+ is presented, whereas the initial excitatory association is independent of context (Bouton, 2004). Since the original excitatory meaning (CS+/US) is not erased by extinction, it can be retrieved following extinction, as evidenced by increased conditional threat responding. In the context of exposure, the retrieval of the excitatory CS+/US association translates to a return of fear and relapse (Vervliet, Hermans, & Craske, 2013).

Several phenomena demonstrate retention of the original excitatory CS+/US association. These include *spontaneous recovery* (Quirk, 2002), which is observed clinically as increasing threat responding with increasing intervals of time since the end of exposure therapy and the next time the phobic stimulus is encountered. For example, an individual who completes treatment for phobia of public speaking will likely have greater threat responding when giving a public speech months after treatment compared to a public speech immediately after the last exposure session. Retention of the CS+/US association is also apparent in *renewal* of threat responding due to a change in context between extinction and extinction retest (Bouton, 1993). Contexts may be exteroceptive cues (e.g., a room, place, environment, or other external background stimuli; Bouton, 1993) and interoceptive cues, such as drug state (Bouton, Kenney, & Rosengard, 1990; Overton, 1985). The clinical translation of context renewal is exemplified by return of fear in a public speaking situation (e.g., a wedding) that differs from the public speaking practiced in exposure therapy (e.g., clinic rooms; Culver, Stoyanova, & Craske, 2011).

A third demonstration of CS+/US retention is *rapid reacquisition*, in which the CS+ and US are re-paired following extinction (Kehoe & Macrae, 1997). Clinically, an individual who undergoes therapy for a phobia of dogs may experience rapid reacquisition if attacked by a dog after completion of exposure therapy. Finally, unsignaled US presentations (without the presence of the CS+) after extinction can lead to a *reinstatement* of threat responding (Rescorla & Heth, 1975). For example, an individual who is treated for specific phobia for dogs after being attacked by a dog may experience reinstatement of fear of dogs following being bitten by a snake. Reinstatement has been long established in animal studies and more recently in human conditioning studies (e.g., Dirikx, Hermans, Vansteenwegen, Baeyens, & Eelen, 2004, 2007; Hermans et al., 2005; LaBar & Phelps, 2005; Norrholm et al., 2006; Van Damme, Crombez, Hermans, Koster, & Eccleston, 2006; Zbozinek, Prenoveau, Liao, Hermans, & Craske, 2015). The current study addresses new ways to mitigate the effects of reinstatement.

There has been little investigation of the effects of a reinstating US that is different from the acquisition US. Yet, reinstatement by a US that differs from the acquisition US would offer a theoretical model for the occasions when clients experience a return of fear following exposure therapy due to an aversive event (e.g., car accident) that differs from the original acquisition event (e.g., social ridicule). In one animal study, a novel US at reinstatement (i.e., klaxon [loud horn]) that differed from the original US during acquisition (i.e., electric shock) reinstated conditional threat responses to the CS+ (Rescorla & Heth, 1975). In one human study, a reinstating US that was different from the acquisition US elicited as great an increase in skin conductance responding to the CS+ as reinstatement with the original US. However, US expectancy increased only for the reinstatement US, regardless of acquisition US (Sokol & Lovibond, 2012). These studies suggest that a reinstating US that differs from the original acquisition US can increase conditional threat responding without increasing expectancy of the acquisition US. The current study evaluated the role of US type at reinstatement (i.e., electric shock, loud scream sound).

Evaluation of CS+ valence in relation to phenomena such as spontaneous recovery has suffered methodological limitations (Dirikx et al., 2004, 2007; Hermans et al., 2005; Zbozinek et al., 2015) or is nonexistent in the case of rapid reacquisition and renewal. However, the more negatively the CS+ is valenced at the end of extinction, the greater the threat responding after reinstatement (Dirikx et al., 2004, 2007; Hermans et al., 2005; Zbozinek et al., 2015). Hermans and colleagues (e.g., Dirikx, et al., 2004) utilized the network model of emotions (Lang, Bradley, & Cuthbert, 1990) to develop the valence-arousal model of reinstatement. In this model, emotions are located on a valence (positive, negative) × arousal (high, low) orthogonal matrix, with fear being located in the negative valence and high arousal quadrant (Lang, Greenwald, Bradley, & Hamm, 1993). Extinction learning decreases arousal towards the CS+, as shown by attenuated skin conductance response (SCR; e.g., Bradley, Cuthbert, & Lang, 1990). However, even though CS+ valence may become less negative by the end of extinction, it typically remains more negative than before acquisition (Dirikx et al., 2004). The combination of increased arousal that is evoked by the arousing properties of the unsignaled US and persistent negative valence of the CS+ is posited to lead to reinstatement of conditional fear responding (Dirikx et al., 2004; Dirikx et al., 2007).

The valence-arousal model of reinstatement raises the possibility that strategies designed to decrease post-extinction negative valence of the CS+ may reduce the effects of reinstatement. Positive mood induction increases positive valence towards a specific stimulus (Erez et al., 2002; Isen & Shalker, 1982). Furthermore, positive mood induction may activate additional neural pathways associated with enhancing extinction learning (e.g., ventromedial/medial prefrontal cortex and anterior cingulate cortex; Phan, Wager, Taylor, & Liberzon, 2002). We predict that positive mood induction may reduce reinstatement effects by decreasing negative valence towards the CS+. A number of methods have been shown to induce positive mood, such as watching positive films (e.g., Gross & Levenson, 1995) and positive imagery training (Holmes, Mathews, Dalgleish, & Mackintosh, 2006; Holmes, Mathews, Mackintosh, & Dalgleish, 2008; Pictet, Coughtrey, Mathews, & Holmes, 2011). We chose positive imagery training given the consistency with which it induces positive mood compared to a stringent comparison condition of positive verbal training (Holmes et al., 2006; Holmes, Lang, & Shah, 2009; Mathews, Ridgeway, & Holmes, 2013; Nelis, Vanbrabant, Holmes, & Raes, 2012).

We hypothesized that positive imagery training would increase positive affect relative to a control condition involving positive verbal training, consistent with prior research (e.g., Holmes, et al., 2006). Second, given that induction of positive mood has been shown to influence valence appraisals of specific stimuli (Erez et al., 2002; Isen & Shalker, 1982), we hypothesized that positive imagery training would decrease CS+ negative valence by the end of extinction training relative to positive verbal training. Third, we hypothesized that positive imagery training would decrease the effects of reinstatement compared to positive verbal training. Furthermore, we evaluated a reinstating US that was the same as the acquisition US (i.e., electric shock) versus different from the acquisition US (i.e., loud scream). We also tested the effects of positive imagery training relative to positive verbal training on spontaneous recovery to test for specificity of effects to reinstatement.

## 2 Methods

### 2.1 Participants

Participants (N = 100) were students from the University of California, Los Angeles, who participated for either 3 course credits, $25 cash, or a combination. Six participants dropped out partway through the study, leaving 94 completers. Participants were 67.3% female; mean age 20.39 (SD = 2.66) years; and 4.3% African-American, 40.4% Asian or Asian-American, 20.2% Caucasian, 22.3% Hispanic or Latino, 7.4% Asian or Asian American and Caucasian, and 5.4% recorded their ethnicity as “other”.

### 2.2 Design

Participants underwent habituation, acquisition, and then were randomized to either positive imagery training (PIT) or positive verbal training (PVT). Following training, all participants underwent extinction. One week later, extinction retest was followed by reinstatement (randomized to either the same US or a different US relative to acquisition) and then reinstatement test. Training Group (PIT, PVT) and Reinstatement US (shock, scream) were between-subjects factors, and CS (CS+, CS−) and Time (First trial of phase, Last trial of phase) were within-subjects factors. Dependent variables included skin conductance response (SCR), eye blink startle reflex (SR), self-report fear, shock US expectancy, self-report CS valence, and positive affect.

## 3 Materials and apparatus

### 3.1 CS and US

The Pavlovian conditioning procedure was programmed using E-Prime 2 Professional Version 2.0.10.353. The CS+ and CS− were images of a Caucasian male and an Asian female with neutral facial expressions (counterbalanced between participants). Facial images were chosen because human faces as CSs may be evolutionarily prepared and result in better conditioning than non-evolutionarily prepared CSs (e.g., lights). In a meta-analysis, Lissek et al. (2005) found preliminary support that using human face CSs resulted in a larger effect size for fear acquisition than non-evolutionarily prepared CSs, but there was no difference in extinction learning. The CSs were displayed on a 21-inch computer monitor for 8 s located 3 feet from the participants at eye level. To maximize CS salience, the CSs covered the entire computer screen when displayed. The CSs were pseudo-randomized with no more than two consecutive presentations of the same CS in a given phase. Inter-trial intervals (ITIs) were randomized to either 25 or 35 s and involved a white screen with a small black fixation cross in the center. Two USs were used: an electric shock and a loud human scream sound. All participants received the shock US during acquisition, and participants were randomized to receive either the shock or scream at reinstatement. The shock US was delivered to the dominant arm bicep using the STMEPM, two LEAD110A (BIOPAC, Inc.), and two Telectrode T716 Ag/AgCl electrodes. The shock consisted of 10 consecutive pulses 0.05 s in duration, totaling 0.5 s. During the acquisition phase, the shock US began 7.5 s after CS+ onset and co-terminated with the CS+. The intensity of the US was determined using a work-up procedure (see Procedures). The scream US was delivered biaurally through stereophonic headphones and lasted 1 s at 96 dB (dB).

### 3.2 Physiological measures

The BIOPAC MP150 hardware unit and AcqKnowledge version 4.2 software (BIOPAC Systems, Inc.) were used to acquire all physiological data.

#### 3.2.1 Skin conductance response (SCR)

SCRs were recorded as a measure of arousal from two EL507 11 mm diameter Ag/AgCl electrodes placed on the distal phalanx of the index and middle fingers of the non-dominant hand (e.g., Bradley et al., 1990). Using a GSR100C amplifier and two LEAD110A, SCR data were sampled at a rate of 31.25 Hz and filtered using an FIR low pass filter with a frequency cutoff fixed at 2 Hz. SCR was calculated as a difference score between the maximum skin conductance value 1–6 s after CS onset minus the mean skin conductance value of the 2 s prior to CS onset. SCR was range-corrected by dividing by the largest SCR for a given participant in a given day. SCRs that were greater than or equal to zero were square root transformed to normalize the data. SCRs less than zero were coded as zero.

#### 3.2.2 Eye blink startle reflex (SR)

SR was measured by electromyography (EMG) orbicularis oculi activity under the left eye using two EL254S 4 mm Ag/AgCl electrodes with the EMG100C amplifier. SR is an indicator of defensive emotional responding and can be considered an index of threat responding to specific cues (Lang et al., 1990). Electrode placement was directly beneath the pupil and 8 mm below the lower eyelid for the first electrode and 1 cm towards the outside of the eye from the first electrode and 8 mm below the lower eyelid for the second electrode. The startle probes (i.e., acoustic startle stimuli delivered to elicit eye blink startle reflexes) consisted of 50 ms, 65 dB bursts of “white noise” with an instantaneous rise time delivered binaurally through stereophonic headphones. Startle probes were randomized to occur, a) 10, 15, or 25 s after onset of the inter-trial interval (i.e., ITI), averaging at 16.25 s, and b) 5 or 6.5 s after CS onset, averaging at 5.75 s. EMG data were sampled at a rate of 2 kHz. The data were filtered using an FIR band pass filter with a low frequency cutoff of 30 Hz and a high frequency cutoff of 1,000 Hz, an IIR band stop filter at line frequency (60 Hz), and the data were smoothed using a smoothing factor of 11 samples using mean value smoothing. SR was calculated as the difference between the absolute maximum EMG level in volts during the 20 ms–150 ms immediately after the startle probe and the mean EMG level in volts during the 200 ms immediately preceding the startle probe. These values were then transformed into T-scores.

### 3.3 Self-report measures

#### 3.3.1 Shock US expectancy

To test explicit learning, participants were instructed to rate “how certain you are that you will receive muscle stimulation [*i*.*e*., *shock*] in the next few moments” using a sliding dial (BIOPAC model TSD115). Participants received 3-s prompts at the beginning of each ITI and CS reminding them to use the expectancy dial. The values ranged from 0 = “Certain no muscle stimulation”, 4.5 = “Uncertain,” and 9 = “Certain muscle stimulation.” Shock US expectancy was calculated as the mean rating 0.5 s before the earliest potential startle probe onset (i.e., 9.5–10 s after ITI onset, 4.5–5 s after CS+ or CS− onset).

#### 3.3.2 Self-report fear

Participants rated “how fearful you are of this image” using a 1–7 scale, where 1 = “Not at all fearful of” and 7 = “Very fearful of.” “This image” refers a small image of the CS+ or CS− in the top-left corner of the computer screen. Fear was measured retrospectively after each threat conditioning phase.

#### 3.3.3 Valence

Participants rated “how positive or negative this image is to you” using a 1–7 scale, where 1 = “Very negative,” 4 = “Neutral,” and 7 = “Very positive.” “This image” refers a small image of the CS+ or CS− in the top-left corner of the computer screen. Self-report valence was measured at the same time points as self-report fear.

#### 3.3.4 Positive and Negative Affect Schedule (PANAS; Watson, Clark, & Tellegen, 1988)

Participants completed the brief 20-item PANAS directly before and after PIT or PVT to measure positive and negative affect in the present moment. Cronbach's α from Watson et al. (1988) was 0.89 and 0.85, respectively, and the correlation between positive and negative affect was −0.15. In the present study, Cronbach's α was 0.93 for positive affect and 0.86 for negative affect; the correlation between positive and negative affect was −0.11 (p = .31). Positive affect was assessed using the positive affect subscale, and negative affect was assessed using the negative affect subscale.

### 3.4 Positive imagery training (PIT) and positive verbal training (PVT)

PIT and PVT involve standardized procedures in which an individual is presented with 100 hypothetical audio scenarios each 10–13 s in duration (e.g. Holmes et al., 2006; Holmes et al., 2009; Nelis et al., 2012). The resolutions to these scenarios are ambiguous until the last word or last few words, but they all end positively (Clarke et al., 2014). For example, “It's your birthday, and your partner reaches over to you with a present. You open it and feel incredibly *happy*.” The ending (in italics) is positive. PIT and PVT only differ in their instructions: PIT participants are trained to imagine each of the scenarios and then to rate the vividness of their mental image, whereas PVT participants are trained to concentrate on the words and meaning of each scenario and then rate how difficult it was to understand the scenarios. In sum, the duration of PIT and PVT was approximately 30 min.

#### 3.4.1 Manipulation check

As a manipulation check of how much each group utilized mental imagery and verbal comprehension, all participants were asked to rate “How much did you find yourself thinking in images (i.e., in mental pictures and sensory impressions) as you were listening to the sentences?” and “How much do you find yourself verbally analyzing the meaning of the sentences as you were listening to them?” Ratings were made using a 1–9 scale, where 1 = “Not at all,” 5 = Half the time,” and 9 = “All the time.”

### 3.5 Procedures

The experiment consisted of two assessments one week apart (i.e., Day 1 and Day 8). On Day 1, participants provided informed consent, and physiological equipment was attached. Participants then engaged in the shock workup procedure. Shocks started at a low intensity and increased to the level a participant considered “uncomfortable but not painful” (i.e., a rating of 6 or 7) using a 0–10 discomfort scale (0 = “Not at all,” 5 = “Moderately,” and 10 = “Very”). Participants were then trained to use the shock US expectancy dial. Next, participants underwent the primary experimental phases: habituation (2 CS+ and 2 CS−), acquisition (8 CS+/US and 8 CS−), training (PIT, PVT), and extinction (8 CS+ and 8 CS−) during Day 1 (see Table 1 for details). On Day 8, physiological equipment was attached, participants were reminded how to use the US expectancy dial, and they commenced extinction retest (2 CS+ and 2 CS−), reinstatement (2 USs), and reinstatement test (2 CS+ and 2 CS−). During the reinstatement phase, participants were randomized to receive either two unsignaled shock USs or two unsignaled scream USs. These occurred randomly either 24 and 75 or 51 and 75 s after onset of the reinstatement phase while a white screen was displayed on the computer.

## 4 Results

### 4.1 Preliminary and baseline analyses

Using one-way ANOVAs, CS Sex (Male, Female) did not significantly impact any dependent measures (ps > 0.14). Also, using chi-squared tests, participant sex and ethnicity did not significantly differ between Training Group (i.e., PIT, PVT) or between Reinstatement US Type (i.e., shock, scream; ps > 0.14). Also, there were no effects involving Training Group or Reinstatement US Type on subjective discomfort of the shock at the end of the workup procedure (M = 6.10, SD = 0.88; ps > 0.55). Thus, baseline variables were not covaried in the analyses below.

As a manipulation check, we evaluated the degree to which participants reported using imagery and verbal comprehension during PIT or PVT. The Training Group (PIT, PVT) × Mentation (Imagery, Verbal Comprehension) mixed model was significant (χ^2^(1) = 122.32, p < .01, f^2^ = 0.63). Simple effects showed that PIT participants reported using significantly more imagery (M = 7.57, SD = 2.02) than verbal comprehension (M = 4.33, SD = 2.53), (χ^2^(1) = 96.62 p < .01). Conversely, PVT participants reported using significantly more verbal comprehension (M = 6.10, SD = 2.53) than imagery (M = 4.18, SD = 2.02), (χ^2^(1) = 33.77, p < .01). Moreover, PIT participants reported using significantly more imagery than PVT participants (χ^2^(1) = 105.32, p < .01), and PVT participants reported using significantly more verbal comprehension than PIT participants (χ^2^(1) = 28.39, p < .01). In sum, these results suggest that participants in the PIT and PVT groups engaged in imagery and verbal comprehension as instructed.

### 4.2 PIT, PVT, and positive affect

**Fig. 1:**
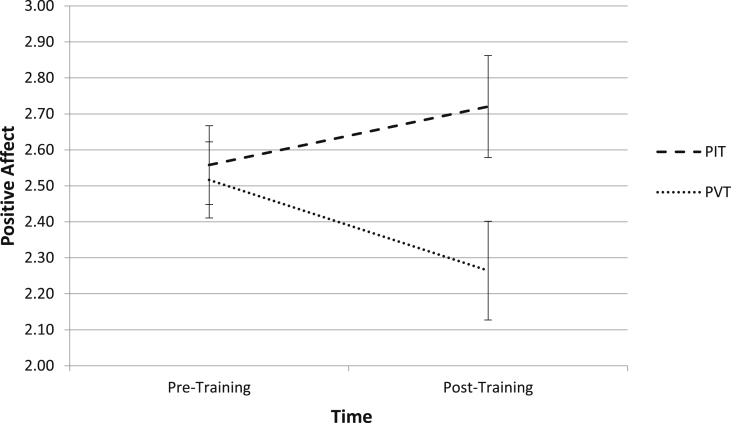
Effect of PIT and PVT on Positive Affect from Before to After Training. PIT = positive imagery training; PVT = positive verbal training. Positive affect was measured on a 1–5 scale using the Positive and Negative Affect Schedule (PANAS; Watson et al., 1988) with higher numbers indicating more positive affect.

The hypothesis that PIT would increase positive affect relative to PVT was analyzed using Training Group (PIT, PVT) × Time (Pre-Training, Post-Training) mixed models with positive affect as the dependent variable. The Training Group (PIT, PVT) × Time (Pre-Training, Post-Training) interaction was significant (χ^2^(1) = 7.86, p < .01, f^2^ = 0.08; see Fig. 1). For PIT, positive affect (i.e., PANAS positive subscale) did not significantly change from pre-training (M = 2.56, SD = 1.04) to post-training (M = 2.72, SD = 1.36), (χ^2^(1) = 2.22, p = .14). However, for PVT, positive affect significantly decreased from pre-training (M = 2.52, SD = 1.01) to post-training (M = 2.27, SD = 1.31), (χ^2^(1) = 6.20, p = .01). At pre-training, positive affect did not differ between PIT and PVT (χ^2^(1) = 0.01, p = .91), whereas at post-training, critically we found that positive affect was significantly higher for PIT than PVT (χ^2^(1) = 5.06, p = .02). In sum, PVT decreased in positive affect, and PIT had higher positive affect than PVT after training (i.e., before extinction).

To test specificity, we examined the effects of PIT and PVT on negative affect (i.e., PANAS negative affect subscale). The Training Group (PIT, PVT) × Time (Pre-Training, Post-Training) interaction was not significant, (χ^2^(1) = 1.25, p = .26), suggesting that the effects of PIT and PVT are specific to positive affect.

### 4.3 Acquisition

To analyze acquisition, we ran Training Group (PIT, PVT) × CS (CS+, CS−) × Time (First Trial of Acquisition, Last Trial of Acquisition) mixed models with SR, shock US expectancy, and self-report fear as dependent variables. Please see Table 2 for descriptive and inferential statistics on acquisition and Table 3 for a summary of threat conditioning results and hypotheses results.^2^ In general, significant differential acquisition was observed for SR, self-report fear, and shock US expectancy (i.e., greater responding to the CS + than the CS−). There was a significant increase in responding to the CS+ from the first to last trial of acquisition for self-report fear and shock US expectancy but not SR. All three measures had significantly higher values for CS+ than CS− at the last trial of acquisition. There was also a significant decrease in responding to the CS− from the first trial of acquisition to the first trial of extinction for SR and shock US expectancy but not self-report fear. There were no effects involving Training Group for any dependent measure (ps > 0.15). In sum, SR, self-report fear, and shock US expectancy showed differential threat responding in acquisition.

### 4.4 Extinction

To analyze extinction, we ran Training Group (PIT, PVT) × CS (CS+, CS−) × Time (First Trial of Extinction, Last Trial of Extinction) mixed models with SR, shock US expectancy, and self-report fear as dependent variables. Please see Table 4 for descriptive and inferential statistics on extinction. In general, significant differential extinction was observed for self-report fear and shock US expectancy. Self-report fear and shock US expectancy yielded significant CS × Time interactions. For both measures, there was a significant decrease in CS+ threat responding from the first to last trial of extinction. For shock US expectancy (but not self-report fear), there was a significant decrease from the first to last trial of extinction for the CS−, as well. We subtracted the shock US expectancy of the first trial of extinction from the last trial of extinction for the CS+ and CS− separately. A dependent samples t-test showed that there was a significantly greater decrease in shock US expectancy for the CS+ (M = −5.449, SD = 3.301) than the CS− (M = −2.562, SD = 3.386), (t(93) = 6.189, p < .01, d = 0.86). Results for SR showed a non-differential decrease in SR from the first to last trial of extinction. There were no effects involving Training Group for any dependent measure (ps > 0.15). In sum, self-report fear and shock US expectancy showed differential extinction, whereas SR showed non-differential extinction.

### 4.5 PIT, PVT, and post-extinction CS valence

**Fig. 2:**
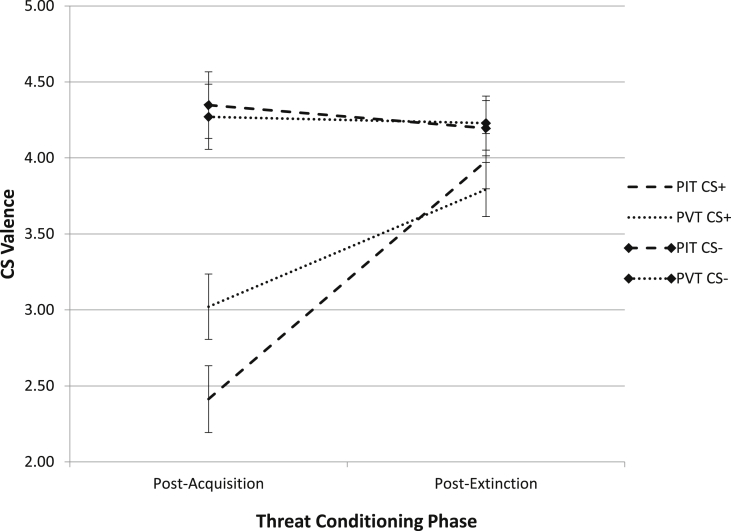
Effect of PIT and PVT Mood Induction on CS+ and CS− Valence. PIT = positive imagery training; PVT = positive verbal training; CS+ is the conditional stimulus associated with the unconditional stimulus (US; during acquisition only); CS− is associated with the absence of the US. CS valence was measured on a 1–7 scale, where 1 = “Very negative,” 4 = “Neutral,” and 7 = “Very positive.”

The hypothesis that PIT would decrease CS+ negative valence more than PVT was analyzed using Training Group (PIT, PVT) × CS (CS+, CS−) × Time (Post-Acquisition, Post-Extinction) mixed models with self-report CS valence as the dependent variable. The three-way Training Group (PIT, PVT) × CS (CS+, CS−) × Time (Post-Acquisition, Post-Extinction) interaction was significant, (χ^2^(1) = 4.29, p = .04. f^2^ = 0.01; see Fig. 2). For CS+, the Training Group × Time interaction was significant, (χ^2^(1) = 6.04, p = .01). Simple main effects showed that, for PIT, CS+ valence significantly increased from post-acquisition (M = 2.41, SD = 1.94) to post-extinction (M = 3.98, SD = 1.45), (χ^2^(1) = 45.95, p < .01). The same pattern occurred for PVT (M = 3.02, SD = 1.89; M = 3.79, SD = 1.42), (χ^2^(1) = 11.63, p < .01). Post-acquisition CS+ valence was subtracted from post-extinction CS+ valence; more positive values represent greater increases in CS+ valence. Critically, an independent samples t-test showed that PIT (M = 1.57, SD = 1.24) increased CS+ valence significantly more than PVT (M = 0.77, SD = 1.59), (t(92) = 2.70, p < .01, d = 0.56). The Training Group × Time interaction was not significant for the CS− (χ^2^(1) = 0.22, p = .64). In sum, as predicted, PIT increased CS+ valence significantly more than PVT, whereas there was no difference between PIT and PVT for the CS−.

### 4.6 Extinction retest

To analyze spontaneous recovery, we ran Training Group (PIT, PVT) × CS (CS+, CS−) × Time (Last Trial of Extinction, First Trial of Extinction Retest) mixed models with SR, shock US expectancy, and self-report fear as dependent variables. Please see Table 5 for descriptive and inferential statistics on extinction retest. In general, significant differential spontaneous recovery was observed for shock US expectancy. Shock US expectancy yielded a significant CS × Time interaction. Both CS+ and CS− significantly increased from the last trial of extinction to the first trial of extinction retest. We subtracted the value at the last trial of extinction from the first trial of extinction retest. A dependent samples t-test for shock US expectancy showed that there was a significantly greater increase for the CS+ (M = 3.68, SD = 3.80) than the CS− (M = 2.32, SD = 3.25), (t(82) = 3.00, p < .01, d = 0.39). Results for SR showed non-differential spontaneous recovery from the last trial of extinction to the first trial of extinction retest. There was also significantly greater SR for the CS+ than CS−. For self-report fear, there was non-differential spontaneous recovery from the end of extinction to after extinction retest. There were no effects involving Training Group for any dependent measure (ps > 0.08). In sum, shock US expectancy showed differential spontaneous recovery, and SR and self-report fear showed non-differential spontaneous recovery. There was also greater SR for the CS+ than CS−.

### 4.7 Reinstatement

The hypothesis that PIT would decrease reinstatement effects compared to PVT was analyzed using Training Group (PIT, PVT) × Reinstatement US Type (Shock, Scream) × CS (CS+, CS−) × Time (Last Trial of Extinction Retest, First Trial of Reinstatement Test) mixed models with SR, shock US expectancy, and self-report fear as the dependent variables. A significant four-way interaction occurred for SR but not for other dependent variables (see Table 6). For PVT who was reinstated with shock, SR to the CS+ significantly increased from the last trial of extinction retest to the first trial of reinstatement test; no such effect was observed for PIT. For PVT who was reinstated with the scream, SR to the CS− significantly increased from the last trial of extinction retest to the first trial of reinstatement test; no such effect was observed for PIT. For PIT who was reinstated with shock, there was significantly higher SR at the last trial of extinction retest for CS+ than CS−; however, there were no differences in SR at the first trial of reinstatement test. Lastly, for PIT who was reinstated with the scream, there was significantly higher SR for the CS+ than CS− at the first trial of reinstatement test. In sum, when reinstated with shock and scream, PVT experienced an increase in SR from before to after reinstatement, but PIT did not. Also, although PIT had significantly higher CS+ than CS− SR before being reinstated with shock, this difference was not present after reinstatement. Conversely, PIT who was reinstated with scream had significantly higher SR for the CS+ than CS− after reinstatement.

For shock US expectancy, the Training Group × US × Time interaction was significant (see Table 7). At the first trial of reinstatement test after being reinstated with shock, PIT had significantly higher shock US expectancy than PVT. For both PIT and PVT, there was a significant increase in shock US expectancy when reinstated with the shock. Using an independent samples t-test, shock US expectancy of the last trial of extinction retest subtracted from the first trial of reinstatement test did not significantly differ between PIT (M = 1.95, SD = 2.95) and PVT (M = 1.21, SD = 2.26), (t(82) = 1.285, p = .20). Thus, though there was an increase in expectancy from before to after the reinstating shock, this did not differ between PIT and PVT. In sum, both PIT and PVT experienced an increase in shock US expectancy, and PIT had greater shock US expectancy after reinstatement than PVT.

Lastly, there were two significant two-way interactions for self-report fear: Training Group × Time and Reinstatement US × Time (see Table 8). For the Training Group × Time interaction, there was a significant decrease in self-report fear for PIT from post-extinction retest to post-reinstatement; no such effect was observed for PVT. For the Reinstatement US × Time interaction, there were no significant simple main effects. In sum, PIT experienced a decrease in self-report fear from before to after reinstatement, but PVT did not.

## 5 Discussion

The present study had several aims. The first aim was to evaluate the effect of positive mood induction on post-extinction CS+ valence (i.e., how positively or negatively participants evaluated the stimulus that formerly predicted electric shock). Second, we evaluated the effect of positive mood induction on threat responding after reinstatement (i.e., experiencing the US [a naturally aversive event] after extinction in absence of the CS+) and whether these effects depended on using the same versus a different US (i.e., electric shock, scream sound) at reinstatement. The results partially supported the main hypotheses.

Positive affect was significantly higher after positive imagery training than positive verbal training. Importantly, positive imagery training led to greater decreases in CS+ negative valence from post-acquisition to post-extinction than did positive verbal training. Most critically, positive imagery training reduced reinstatement threat responding more than positive verbal training. Under typical reinstatement circumstances (i.e., same US at reinstatement as in acquisition), eye blink startle reflex increased from before to after reinstatement for positive verbal training, whereas startle did not change for positive imagery training; in fact, positive imagery training had significantly higher startle for the CS+ than CS− before reinstatement but had no such difference after reinstatement. When reinstated with a novel US, startle increased for the CS− with positive verbal training but not positive imagery training. Conversely, there was significantly higher startle for the CS+ than CS− after reinstatement with positive imagery training but not positive verbal training. Thus, it seems that a novel reinstating US can result in greater startle during safety signals (i.e., CS−) when positive affect is lower before extinction (i.e., positive verbal training) but greater startle during danger signals (i.e., CS+) when positive affect is higher before extinction (i.e., positive verbal training). However, because the literature on the effects of a novel reinstating US on threat responding is sparse and because we had no a priori hypotheses explicating the effects of mood induction on threat responding with a novel reinstating US, replication is needed before conclusions can be drawn on this matter.

Furthermore, self-report fear significantly decreased from before to after reinstatement for positive imagery training but remained unchanged for positive verbal training. Interestingly, when reinstated with shock, there was greater shock US expectancy after reinstatement for positive imagery training than positive verbal training. Thus, compared to positive verbal training, positive imagery training led to less fear and startle reflex despite elevated expectancy of being shocked after reinstatement.

In general, the results partially support the valence-arousal model of reinstatement. However, there are also other models of reinstatement that have received support. The valence-arousal model of reinstatement extends an arousal theory – which posits that an unsignaled US elicits physiological arousal similar to the arousal experienced during fear acquisition and the arousal acts as an internal retrieval cue of the excitatory CS+/US association (Haroutunian & Riccio, 1979) – by including valence as an additional dimension. According to the valence-arousal model of reinstatement, we would expect residual CS+ negative valence from the end of extinction to be combined with a reinstating US, which presumably increases arousal. However, no study has decisively tested changes in both CS+ valence and arousal with reinstatement. Furthermore, because the valence-arousal model of reinstatement has not been tested across multiple contexts, it is unclear how it relates to the context-dependent theory, which posits that fear to the extinguished CS+ occurs only when the US is reinstated in the same context in which the CS+ is tested (Bouton, Westbrook, Corcoran, & Maren, 2006). Though the valence-arousal model of reinstatement suggests that an increase in arousal would be combined with residual negative valence to produce fear, it does not specify whether the source of the arousal (i.e., the reinstating US) needs to be the same US as from acquisition. The results suggest that changes in self-report fear are not dependent on the reinstating US, though changes in startle reflex and shock US expectancy are. Thus, it remains unclear how the source of arousal affects reinstatement.

The results of this study have significant implications for exposure therapy for anxiety disorders. Return of fear following exposure therapy is a major challenge faced by clinicians in the treatment of anxiety disorders. The current results suggest that positive mood induction – in this case, with the use of imagery – before extinction reduces the effects of reinstatement on one measure of explicit fear (i.e., self-report fear) and one measure of implicit threat responding (i.e., startle reflex). Consequently, engaging in positive mood induction with imagery before conducting exposures may reduce return of fear following exposure therapy that occurs due to unpredicted aversive events. In accordance with Bouton et al. (2006), the benefits of positive mood induction may be confined to the reinstating context, though this has yet to be tested.

Positive imagery training as used in the current experiment may not be a pragmatic option in a therapy setting (e.g., time constraints, access to computer). However, there are numerous other mental imagery therapeutic techniques that could be developed swiftly in a session in line with the overall formulation and target for that client (Holmes & Mathews, 2010). The method of positive imagery mood induction could readily be idiosyncratic, chosen collaboratively with the client, and even tailored to best match the type of CS. For example, the therapist might ask the client to imagine the positive aspects of an upcoming positive event (e.g., if the client is excited about attending a professional sporting event, imagining watching the teams play, eating and drinking from the concession stand, the cheering of the crowd, being with family/friends). Non-imagery-based mood induction techniques prior to exposure may also work, such as having a snack, reading a book, or engaging in a positive activity on the client's phone (e.g., playing a game, watching videos, reading the news, calling a family member/friend).

There were several limitations of the present study. First, positive imagery training and positive verbal training were the methods chosen for mood induction. As such, the positive imagery group was trained to use imagery, and positive verbal training was trained to use verbal comprehension. While a procedure involving both imagery and positive information was more effective than its verbal counterpart, we cannot conclude whether the effects on CS+ valence and reinstatement were the result of changes in positive affect, the type of mentation technique used (i.e., imagery, verbal), or the combination of both. Related is the limitation that there was no group which did neither positive imagery training nor positive verbal training, (e.g. neutral imagery training, attention control). Second, the present study only measured explicit self-report CS valence. Future studies would benefit from implicit measures of valence, such as postauricular reflex (Benning, Patrick, & Lang, 2004; Sandt et al., 2009) or implicit attitudes (Vasey, Harbaugh, Buffington, Jones, & Fazio, 2012). Third, self-report fear was measured after extinction retest. This means that participants experienced two non-reinforced trials of the CS+ and CS− prior to making fear ratings. These trials likely resulted in extinction learning that could have lowered fear ratings, thus underestimating spontaneous recovery measured via fear. Fourth, our self-report measures of fear and valence included a small image of the CS on screen with the text of the rating scale and fear/valence question. This small image likely reduced salience of the CS when making these ratings and may have possibly resulted in some extinction. Fifth, the findings model a one-week lapse in time for spontaneous recovery and only a few minutes lapse for reinstatement, as well as only one session of extinction learning. It is unclear how these results would extend over longer periods of time or multiple sessions of extinction/exposure, which would be relevant for treatment of anxious individuals.

While there was a fairly consistent pattern on startle reflex and self-report fear at reinstatement, the results are not fully robust across all indices. Indeed, although the various measures of “fear” as a construct often covary (Craske, Hermans, & Vansteenwegen, 2006), there is often discrepancy between the various measures of “fear.” This can result for many reasons (e.g., measurement error, differences in sensitivity; Boddez et al., 2013), but it may be an indication that each measure captures a different aspect of “fear.” Startle reflex is a measure of defensive responding that changes based on emotional valence and is independent of arousal (Lang et al., 1990). Skin conductance is a measure of arousal (Bradley et al., 1990; Cook, Hawk, Davis, & Stevenson, 1991; Greenwald, Cook, & Lang, 1989; Manning & Melchiori, 1974; Winton, Putnam, & Krauss, 1984), which does not necessarily covary with the startle reflex (Lang et al., 1990; Soeter & Kindt, 2010). US expectancy is not directly a measure of “fear,” but rather a measure of associative learning (i.e., the CS/US relationship). There may be an emotion that covaries with US expectancy (e.g., excitement if the US is appetitive, fear if the US is aversive), but US expectancy likely does not directly measure these emotions. Lastly, self-report fear is a measure of the explicit emotion of being afraid and may not include other threat-relevant responding (LeDoux, 2014).

Future studies could also evaluate the effects of CS+ valence and positive mood induction on other exemplars of retrieval of excitatory associations following extinction, such as context renewal and rapid reacquisition. It is conceivable that CS+ valence may influence these phenomena. As with reinstatement, rapid reacquisition and context renewal may both involve increases in arousal: rapid reacquisition through experiencing the US (paired with the CS+), and context renewal through entering a novel context (King & Williams, 2009). The combination of increased arousal and the negative valence toward the CS+ that persists after extinction could enhance both rapid reacquisition and context renewal. Hence, methods for reducing CS+ negative valence may attenuate both processes. Furthermore, generalizability of the findings to a clinically anxious sample awaits further investigation.

In conclusion, the present study suggests that more positive mood before extinction reduces post-extinction CS+ negative valence and reduces threat responding after reinstatement. This finding raises the possibility that a positive mood induction before conducting an exposure therapy session could reduce subsequent return of fear.

## Figures and Tables

**Table 1:** Overview of the experimental procedures with order of phases listed in italics and order within phases listed vertically.

Day 1						
Habituation	Acquisition	PIT or PVT	Extinction	Extinction Retest	Reinstatement	Reinstatement Test
2 CS+, 2 CS−, 4 ITIs	8 CS+ with 8 US, 8 CS−, 16 ITIs	PANAS	8 CS+, 8 CS−, 16 ITIs	2 CS+, 2 CS−, 4 ITIs	2-min ITI with 2 USs	2 CS+, 2 CS−, 4 ITIs
Valence & Fear Ratings	Valence & Fear Ratings	Training	Valence & Fear Ratings	Valence & Fear Ratings	Valence & Fear Ratings	Valence & Fear Ratings
		PANAS				

Note: PIT refers to positive imagery training, PVT refers to positive verbal training, CS+ is the conditional stimulus that, during acquisition, is paired with the unconditional stimulus (US; in acquisition, the US was electric shock; at reinstatement, participants were randomized to either electric shock or a scream sound as the US), CS− is associated with the absence of the US, ITIs are inter-trial intervals (i.e., the time between presentations of the CS+ and CS−), PANAS is the Positive and Negative Affect Schedule, and valence ratings are how positively or negatively participants evaluate the CS+ and CS−.

**Table 2:** Acquisition.

		First trial acquisition	Last trial acquisition	2-way^b^	Simple main effect	Main effect	χ^2^^a^	p	f^2^
SR	CS+	49.62 (7.16)	49.35 (8.76)	TG × CS			1.71	0.19	
	CS−	50.59 (9.10)	47.58 (5.88)	TG × Time			0.58	0.45	
				**CS** × **Time**			**8.34**	**<.01**	**0.03**
					**CS @ Last Trial Acquisition**		**6.94**	**<.01**	
					**Time @ CS−**		**18.90**	**<.01**	
						TG	0.22	0.64	
Shock US Expectancy	CS+	3.11 (3.04)	7.20 (3.66)	TG × CS			0.43	0.51	
	CS−	4.60 (3.22)	2.25 (3.42)	TG × Time			0.09	0.76	
				**CS** × **Time**			**94.54**	**<.01**	**0.34**
					**CS @ First Trial Acquisition**		**10.53**	**<.01**	
					**CS @ Last Trial Acquisition**		**110.29**	**<.01**	
					**Time @ CS+**		**25.27**	**<.01**	
					**Time @ CS−**		**76.27**	**<.01**	
						TG	0.59	0.44	
Self-Report Fear	CS+	3.22 (1.71)	4.52 (1.65)	TG × CS			0.05	0.82	
	CS−	3.15 (1.57)	2.93 (1.74)	TG × Time			0.08	0.78	
				**CS** × **Time**			**28.23**	**<.01**	**0.11**
					**CS @ Last Trial Acquisition**		**61.85**	**<.01**	
					**Time @ CS+**		**41.46**	**<.01**	
						TG	0.94	0.33	

Note: TG = Training Group, CS+ is the conditional stimulus associated with the unconditional stimulus (US) during acquisition but not extinction, CS− is associated with the absence of the US, and SR is startle reflex.

a Degrees of freedom for all analyses = 1.

b There were no significant 3-way interactions.

**Table 3:** Summary of hypotheses and threat conditioning outcomes.

Hypothesis	Measure	Supported?
Mood Induction	Positive Affect	Yes
Extinction CS Valence	CS+ and CS− Valence	Yes
Reinstatement	Startle Reflex	Yes
	Shock US Expectancy	No
	Self-Report Fear	Yes

Threat conditioning phase	Measure	Differential Effect?^a^	Non-differential Effect?^b^
Acquisition	Startle Reflex	**Yes**	–
	Shock US Expectancy	**Yes**	–
	Self-Report Fear	**Yes**	–
Extinction	Startle Reflex	No	**Yes**
	Shock US Expectancy	**Yes**	–
	Self-Report Fear	**Yes**	–
Spontaneous Recovery	Startle Reflex	No	**Yes**
	Shock US Expectancy	**Yes**	–
	Self-Report Fear	No	**Yes**

Note: CS+ is the conditional stimulus associated with the unconditional stimulus (US), and CS− is associated with the absence of the US.

a “Differential effect” refers to a difference in CS+ and CS− threat responding. This difference was in the expected direction for each phase.

b “Non-differential effect” refers to no difference in CS+ and CS− threat responding. All effects were in the expected direction for each phase.

**Table 4:** Extinction.

			First trial extinction	Last trial extinction	2-way^b^	Simple main effect	Main effect	χ^2^^a^	p	f^2^
SR	PIT	CS+	54.76 (17.89)	48.38 (8.94)	TG × CS			0.23	0.63	
		CS−	54.60 (18.88)	48.38 (7.72)	TG × Time			0.00	0.98	
	PVT	CS+	52.91 (11.79)	47.46 (7.36)	CS × Time			0.20	0.65	
		CS−	53.93 (14.05)	46.82 (5.58)			TG	0.23	0.63	
							CS	0.00	0.98	
							**Time**	**54.24**	**<.01**	
Shock US Expectancy	PIT	CS+	6.48 (3.49)	1.26 (2.54)	TG × CS			1.74	0.19	
		CS−	2.81 (3.11)	0.93 (2.10)	TG × Time			3.59	0.06	
	PVT	CS+	6.60 (3.09)	0.90 (2.06)	**CS** × **Time**			**32.70**	**<.01**	**0.11**
		CS−	3.99 (2.95)	0.69 (2.03)		**CS @ First Trial Extinction**		**78.72**	**<.01**	
						**Time @ CS+**		**241.42**	**<.01**	
						**Time @ CS−**		**54.68**	**<.01**	
							TG	0.15	0.69	
Self-Report Fear	PIT	CS+	4.64 (1.57)	3.02 (1.47)	TG × CS			0.98	0.32	
		CS−	3.10 (1.69)	2.95 (1.61)	TG × Time			0.37	0.55	
	PVT	CS+	4.41 (1.74)	3.16 (1.61)	**CS** × **Time**			**19.65**	**<.01**	**0.07**
		CS−	2.75 (1.79)	2.61 (1.54)						
						**CS @ First Trial Extinction**		**62.11**	**<.01**	
						**Time @ CS+**		**48.69**	**<.01**	
							TG	0.72	0.40	

Note: TG = Training Group, CS+ is the conditional stimulus associated with the unconditional stimulus (US) during acquisition but not extinction, CS− is associated with the absence of the US, and SR is startle reflex.

a Degrees of freedom for all analyses = 1.

b There were no significant 3-way interactions.

**Table 5:** Extinction retest.

			Last trial extinction	First trial extinction retest	2-way^b^	Simple main effect	Main effect	χ^2^^a^	p	f^2^
SR	PIT	CS+	48.35 (9.07	51.89 (8.12)	TG × CS			1.44	0.23	
		CS−	48.31 (7.86)	51.09 (8.12)	TG × Time			2.33	0.13	
	PVT	CS+	47.53 (7.43)	52.89 (8.80)	CS × Time			3.94	0.09	
		CS−	46.82 (5.65)	49.76 (7.00)			TG	0.66	0.42	
							**CS**	**5.62**	**0.02**	
							**Time**	**43.97**	**<.01**	
Shock US Expectancy	PIT	CS+	1.42 (2.68)	4.65 (3.38)	TG × CS			0.01	0.94	
		CS−	0.89 (2.11)	2.89 (2.83)	TG × Time			3.01	0.08	
	PVT	CS+	0.94 (2.10)	5.04 (3.37)	**CS** × **Time**			**5.91**	**0.02**	**0.02**
		CS−	0.72 (2.07)	3.34 (2.75)						
						**CS @ Extinction Retest**		**16.65**	**<.01**	
						**Time @ CS+**		**96.17**	**<.01**	
						**Time @ CS−**		**38.39**	**<.01**	
							TG	0.14	0.71	
Self-Report Fear	PIT	CS+	3.13 (1.46)	3.63 (1.84)	TG × CS			0.06	0.80	
		CS−	3.08 (1.58)	3.29 (1.69)	TG × Time			0.49	0.48	
	PVT	CS+	3.20 (1.62)	3.59 (1.76)	CS × Time			1.71	0.19	
		CS−	2.68 (1.54)	2.64 (1.51)			TG	0.87	0.35	
							CS	14.12	<0.01	
							**Time**	**4.17**	**0.04**	

Note: TG = Training Group, CS+ is the conditional stimulus associated with the unconditional stimulus (US) during acquisition but not extinction, CS− is associated with the absence of the US, and SR is startle reflex.

a Degrees of freedom for all analyses = 1.

b There were no significant 3-way interactions.

**Table 6:** Reinstatement – SR.

			Last trial extinction retest	First trial reinstatement test	4-way	Simple 3-way	Simple 2-way	Simple main effect	χ^2^^a^	p	f^2^
PIT	Shock	CS+	52.61 (10.89)	51.62 (8.02)	**TG × US × CS × Time**				**13.87**	**<0.01**	**0.06**
		CS−	49.51 (7.49)	52.04 (10.34)		**TG × CS × US @ Extinction Retest**			**7.04**	<**0.01**	
	Scream	CS+	50.44 (5.57)	52.73 (8.53)			**TG × CS @ Shock Extinction Retest**		**6.53**	**0.01**	
		CS−	51.72 (7.63)	49.64 (6.22)				**CS @ PIT Shock Extinction Retest**	**5.18**	**0.02**	
PVT	Shock	CS+	48.41 (5.02)	51.53 (8.27)		**TG × CS × Time @ Shock**			**7.72**	**<0.01**	
		CS−	50.56 (6.79)	49.07 (5.3)			**CS × Time @ PVT Shock**		**4.43**	**0.04**	
	Scream	CS+	48.55 (6.87)	49.11 (6.88)				**Time @ PVT Shock CS+**	**4.03**	**<0.05**	
		CS−	47.32 (4.08)	50.77 (10.76)		**TG × CS × Time @ Scream**			**6.21**	**0.01**	
							**TG × CS @ Scream Reinstatement Test**		**5.32**	**0.02**	
								**CS @ PIT Scream Reinstatement Test**	**3.91**	**<0.05**	
							**TG × Time @ Scream CS−**		**7.2**	**<0.01**	
								**Time @ PVT Scream CS−**	**6.61**	**0.01**	

Note: TG = Training Group, CS+ is the conditional stimulus associated with the unconditional stimulus (US) during acquisition but not extinction, CS− is associated with the absence of the US, SR is startle reflex, and US on the right portion of the table is Reinstatement US Type.

a Degrees of freedom for all analyses = 1.

**Table 7:** Reinstatement - shock US expectancy.

			Last trial extinction retest	First trial reinstatement test	4-way	3-way	Simple 2-way	Simple main effect	Main effect	χ^2^^a^	p	f^2^
PIT	Shock	CS+	2.92 (3.61)	5.94 (3.01)	TG × US × CS × Time					0.13	0.72	
		CS−	1.74 (2.65)	4.64 (3.03)		**TG** × **US** × **Time**				**5.44**	**0.02**	**0.17**
	Scream	CS+	2.05 (2.90)	2.58 (2.44)			**TG** × **US @ Reinstatement Test**			**8.82**	**<.01**	
		CS−	1.37 (2.42)	1.26 (2.01)				**TG @ Shock Reinstatement Test**		**5.38**	**0.02**	
PVT	Shock	CS+	3.37 (3.24)	4.28 (1.93)				**US @ PIT Reinstatement Test**		**23.34**	**<.01**	
		CS−	1.39 (2.27)	3.59 (2.32)			**TG** × **Time @ Shock**			**5.02**	**0.03**	
	Scream	CS+	3.07 (3.29)	3.91 (3.43)				**Time @ PVT Shock**		**10.89**	**<.01**	
		CS−	2.44 (2.81)	3.45 (2.83)				**Time @ PIT Shock**		**51.28**	**<.01**	
									**CS**	**17.71**	**<.01**	

Note: TG = Training Group, CS+ is the conditional stimulus associated with the unconditional stimulus (US) during acquisition but not extinction, CS− is associated with the absence of the US, and US on the right portion of the table is Reinstatement US Type.

a Degrees of freedom for all analyses = 1.

**Table 8:** Reinstatement – self-report fear.

			Last trial extinction retest	First trial reinstatement test	2-way	Simple main effect	Main effect	χ^2^^a^	p	f^2^
PIT	Shock	CS+	3.61 (2.06)	2.87 (2.01)	TG × US			1.35	0.25	
		CS−	3.35 (1.77)	3.04 (1.58)	TG × CS			3.33	0.07	
	Scream	CS+	3.88 (1.45)	3.71 (1.45)	**TG** × **Time**			**6.31**	**0.01**	**0.01**
		CS−	3.24 (1.68)	3.24 (1.52)		**Time @ TG**		**4.25**	**0.04**	
PVT	Shock	CS+	3.95 (1.66)	3.71 (1.55)	US × CS			0.75	0.39	
		CS−	2.95 (1.40)	3.14 (1.46)	**US** × **Time**			**4.29**	**0.04**	**0.01**
	Scream	CS+	3.26 (1.81)	3.52 (1.75)			**CS**	**18.93**	**<.01**	
		CS−	2.35 (1.58)	3.09 (1.59)						

Note: TG = Training Group, US = Reinstatement US Type, CS+ is the conditional stimulus associated with the US during acquisition but not extinction, and CS− is associated with the absence of the US.

a Degrees of freedom for all analyses = 1.

## Footnotes

1 Following premises set forth by LeDoux (2014), we will use the term “threat responding” to encompass both the conscious, emotional experience of being afraid and the unconscious, biological responses. The term “fear” conditioning” will be replaced by “threat conditioning.” The term “fear” will henceforth refer to the conscious emotion of being afraid.

2 Though skin conductance response (SCR) followed expected differential fear acquisition and extinction, there were no effects involving Training Group or Reinstatement US Type at either extinction retest or reinstatement. Thus, to reduce complexity, SCR analyses are not reported.
